# Prevalence and factors associated with carbapenem-resistant Enterobacterales (CRE) infection among hematological malignancies patients with CRE intestinal colonization

**DOI:** 10.1186/s12941-023-00554-6

**Published:** 2023-01-10

**Authors:** Xia Chen, Ximao Wen, Zhiping Jiang, Qun Yan

**Affiliations:** 1 Department of Clinical Laboratory, Xiangya Hospital, Central South University, 87 Xiangya Road, Changsha, 410008 Hunan China; 2 Infection Control Center, Xiangya Hospital, Central South University, 87 Xiangya Road, Changsha, 410008 Hunan China; 3 Department of Hematolology, Xiangya Hospital, Central South University, 87 Xiangya Road, Changsha, 410008 Hunan China; 4 National Clinical Research Center for Geriatric Disorders, Xiangya Hospital, Central South University, 87 Xiangya Road, Changsha, 410008 Hunan China

**Keywords:** Carbapenem-resistant Enterobacterales, Subsequent infection, Hematological malignancy, Associated factors, Rectal colonization

## Abstract

**Background:**

Knowledge about the prevalence, factors and mortality associated with subsequent carbapenem-resistant Enterobacterales (CRE) infection among hematological malignancies (HM) patients colonized with CRE is limited.

**Methods:**

HM patients were screened for rectal CRE. A retrospective case–control study of subsequent CRE infection among HM patients colonized with CRE was conducted between January 1st, 2020 and January 31st, 2022. Cases were defined as CRE colonized patients with subsequent infection and controls were those without infection. Bacterial identification was performed using MALDI Biotyper and antimicrobial susceptibility testing of strains was carried out using the VITEK 2 system or standard broth microdilution method. Logistic analysis was used for analyzing associated factors and Kaplan–Meier method was used for survival estimates.

**Results:**

A total of 953 HM patients were screened for rectal CRE and 98 (10.3%, 98/953) patients were colonized with CRE. Among the 98 colonized patients, 18 (18.4%, 18/98) patients developed subsequent infection. Most of the colonizing CRE isolates were *Klebsiella pneumoniae* (50.0%, 27/54), followed by *Escherichia coli* (27.8%, 15/54) and *Enterobacter cloacae* (9.3%, 5/54). As for the subsequent infecting CRE isolates, the dominated species was *K. pneumoniae* (55.6%, 10/18), followed by *E. coli* (33.3%, 6/18) and others (11.2%, 2/18). Receiving proton pump inhibitors and admission to ICU (*P* < *0.05*) were the associated factors. Patients with subsequent CRE infection had significant higher mortality (33.3% vs 2.8%, P = 0.001) and shock was an associated factor (P = 0.008).

**Conclusions:**

*Klebsiella pneumoniae* was the dominate colonizing species and subsequent infecting species among HM patients with CRE colonization. Receiving proton pump inhibitors and admission to ICU increased the risk of subsequent CRE infection among CRE colonized HM patients. Implementing strict infection control measures targeting those high- risk patients may prevent subsequent CRE infection.

## Background

Carbapenem-resistant Enterobacterale (CRE) infection has become a serious global public health threat and produced considerable clinical and epidemiological challenges with high morbidity and costs, especially in immunocompromised patients [1–3]. Hematological malignancies (HM) patients are usually immunocompromised and at high risk for infections, particularly bloodstream infections (BSI) [4]. Carbapenem-resistant Enterobacterale (CRE) bloodstream infections among hematological malignancies patients have been reported increasingly [5]. CRE is resistant to first-line antimicrobial agents which are recommended for empiric antibiotic therapy for fever in neutropenic patients [6, 7]. The mortality rate of HM patients with CRE infection is ranging from 45.6 to 100% [8–10]. The high mortality may be due to the prolonged neutropenia caused by their underlying malignancies. Moreover, the high dose chemotherapy and mucosal barrier damage may make HM patients prone to infection [7, 8].

Screening CRE rectal colonization among high-risk patients has become an important prevention measure for CRE infection [11, 12]. Studies have revealed high infection rate among carriers. Cattaneo et al. revealed 15.9% (23/144) hematological patients developed a BSI caused by the same previously identified intestinal colonizing pathogen [13]. About 30% hematopoietic stem cell transplant recipients with multidrug-resistant bacteria (MDR) gram-negative bacteria (GNB) intestinal colonization developed subsequent infection [14]. Thus, identifying the factors associated with subsequent infection among MDR bacteria carriers is important for preventing infection. History of carbapenems use and immunocompromise were identified as risk factors associated with carbapenem-resistant gram-negative bacteria infection after colonization among ICU patients [11]; gastrointestinal injury, tigecycline exposure, carbapenem resistance score, high-risk disease and mucositis were related to subsequent CRE infection among colonized patients with general hematological disease [15, 16]. The hematological department in our hospital is the provincial referral center serving a large population of hematological malignancies patients, and it has been identified as one of the units with high CRE infection rate [17].

However, limited data is available regarding factors associated with subsequent CRE infection among HM patients with CRE colonization. It is of great value to explore the factors triggering the translocation of CRE from the gut to infection among HM patients, to better apply infection control measures to reduce the mortality. Therefore, this study aimed to analyze the prevalence, factors and mortality associated with subsequent CRE infection following CRE rectal colonization among HM patients.

## Methods

### Study design, setting and patients

This retrospective case–control study was carried out between January 1st, 2020 and January 31st, 2022 at a 3500-beds tertiary university hospital which has an annual admission of more than 130,000 inpatients in Central-south of China. Hospitalized patients in Hematology Department were screened for rectal CRE colonization by using stool or rectal swabs upon admission and weekly by methods as previous described [18]. Hematological Malignancies patients with CRE rectal colonization were included for further study. Patients who had a CRE infection prior to positive rectal screening test; patients with diagnosis other than hematological malignancies; patients with subsequent infection caused by different bacterial species other than rectal CRE were excluded.

Cases were defined as CRE colonized patients who developed clinical CRE infection with the same species as colonizing CRE after 24 h of positive screening test [19]. Controls were randomly selected from the rest uninfected CRE rectal colonized HM patients with a 2:1 ratio after controlling other potential confounders, such as age, sex and department. Clinical CRE infection was defined as CRE isolated in relevant infection sites and having the signs and symptoms meet the criteria of the corresponding infection definition. Bloodstream infection was diagnosed in patients with positive blood cultures and clinical manifestations. Diagnosis of pneumonia, urinary tract infection, intra-abdominal infection, infectious diarrhea, skin and soft tissue infection, and intracranial infection was based on the USA SIS/IDS/ATS guidelines [20–25].

### Bacterial identification and antimicrobial susceptibility testing

CRE were enterobacterales resistant to at least one of the carbapenems, including imipenem, meropenem, and ertapenem. Bacterial identification was performed by MALDI Biotyper (Bruker, Germany). Antimicrobial susceptibility tests were carried out by Vitek2 (bioMerieux, France) except colistin susceptibility testing which was performed by standard broth microdilution method (Bio-kont, China). *Escherichia coli* strain ATCC 25922 was used for the quality control. Results were interpreted according to the Clinical and Laboratory Standards Institute (CLSI) for all the antimicrobial agents except tigecycline [26], which were interpreted using the US Food and Drugs Administration (FDA) breakpoints [27].

### Factors associated with subsequent CRE infection

Variables were collected from the electronic medical records retrospectively, including: sex, age, length of hospital stay, hospital transfer, admission to intensive care unit (ICU) after identifying CRE colonization, prior hospitalization, diabetes mellitus, solid tumor, pneumonia, liver disease, gastritis, shock, diarrhea, sever neutropenia, central venous catheter, arterial catheter, endotracheal intubation, mechanical ventilation, urinary catheter, nasogastric tube and hematopoietic stem cell transplantation, drug exposure within 4 weeks before rectal CRE detected including proton pump inhibitors (PPIs), glucocorticoid, cephalosporins, carbapenems, aminoglycosides, fluoroquinolones, β-lactam/β-lactamase inhibitors, glycopeptides (including vancomycin and telicoplanin), tigecycline, macrolides, sulfamethoxazole and trimethoprim and antiviral agent and antifungal agents. Variables were compared between cases and controls to identify associated factors for subsequent CRE infection.

### Mortality for subsequent CRE infection

Mortality was observed with a 90-day follow up after positive rectal CRE screening test. Mortality was defined as death related to subsequent CRE infection which was confirmed by positive culture of blood or sterile body fluids, in the absence of other confounding factors [11]. CRE colonized HM Patients with subsequent infection were further divided into subgroups of survival group and mortality group. Then associated factors for mortality of subsequent CRE infection cases were assessed by comparison between those two groups.

### Statistical analysis

All data were analyzed by using SPSS version 26.0 software (IBM corporation, USA). The normality of data distribution was analyzed using the Shapiro–Wilk test. Continuous variables were presented as mean ± SD if normally distributed, or as median and interquartile range (IQR) if non-normally distributed. Categorical variables were compared by Chi-square test or Fisher’s exact test, and continuous variables were analyzed by Student’s t test or the wilcoxon rank-sum test, as appropriate. Variables with *P*-value < 0.05 were checked for multicollinearity and VIF values and then included in a logistic regression model with odds ratio (OR) and 95% confidence intervals (95% CI), in which a *P*-value < 0.05 was defined as statistically significant. A survival analysis of patients with CRE rectal colonization was performed by the Kaplan–Meier method.

## Results

### Patient cohort

**Fig. 1:**
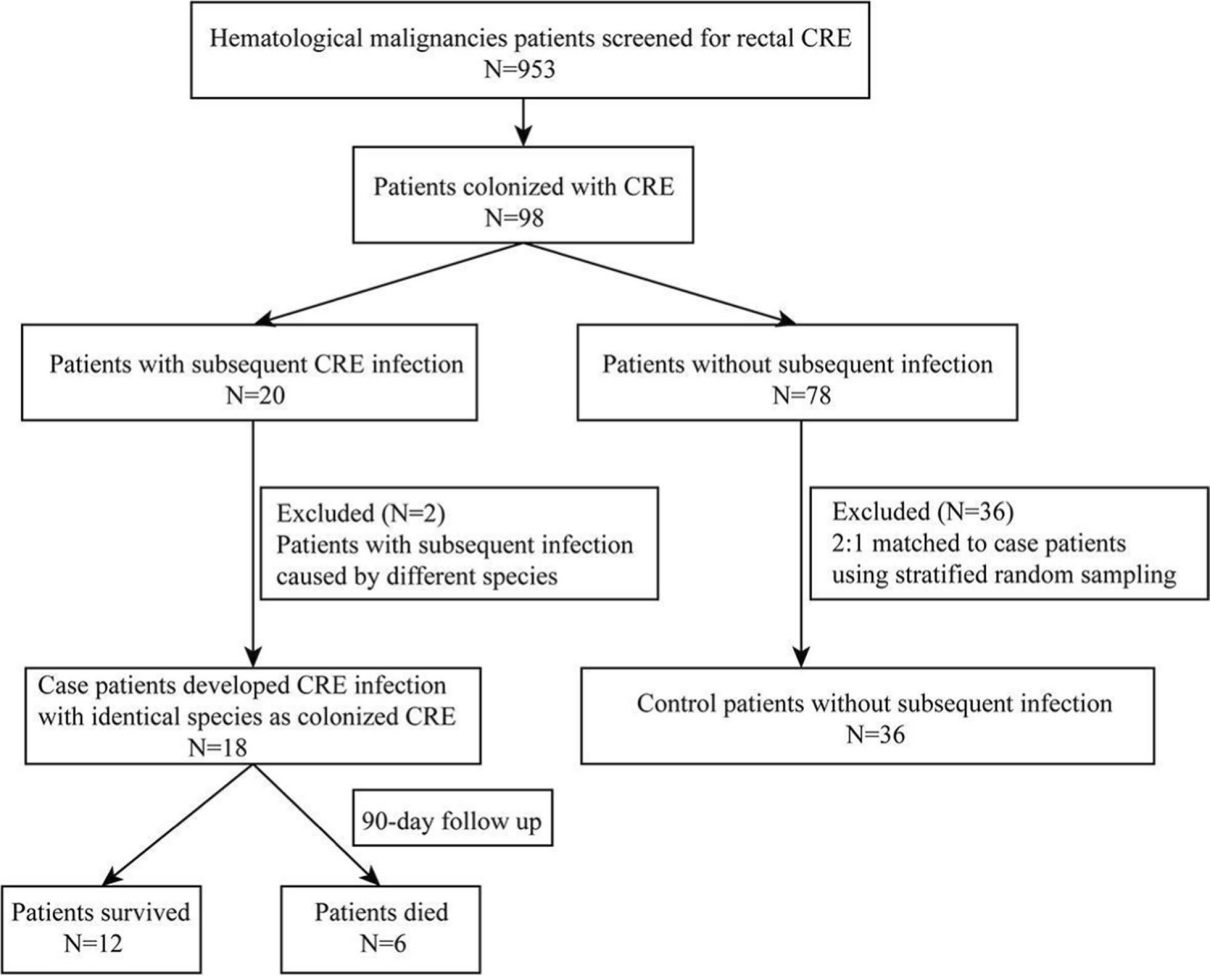
Flowchart of the study design

During the study period, a total of 953 HM patients were screened for rectal CRE and 98 (10.3%, 98/953) were colonized with CRE. Among those 98 colonized HM patients, 18 (18.4%, 18/98) patients had subsequent CRE infection with the same bacterial species as the rectal CRE. There were 36 non-infected HM patients colonized with CRE matched to the cases as controls (Fig. 1). The total 54 studied patients included 13 Acute Lymphoblastic Leukemia (ALL) patients, 26 Acute Myeloid Leukemia (AML) patients, 11 Multiple Myeloma (MM) patients, 2 Lymphoma patients and 2 patients with other diagnosis of HM.

### Species distribution and antimicrobial susceptibility patterns

Among the 54 patients, 27 (50.0%, 27/54) were colonized by *Klebsiella pneumoniae*, 15 (27.8%, 15/54) by *Escherichia coli*, 5 (9.3%, 5/54) by *Enterobacter cloacae* and 7 (12.9%, 7/54) by others. For the case group, the dominated subsequent infecting bacterial species was *K. pneumoniae* (55.6%, 10/18), followed by *E. coli* (33.3%, 6/18) and others (11.2%, 2/18). The majority subsequent clinical infections were bloodstream infections (83.3%, 15/18), and most commonly caused by *K. pneumoniae* (53.3%, 8/15), followed by *E. coli* (40.0%, 6/15). Other 3 clinical infections were pneumonia.

The antimicrobial susceptibility profile of subsequent infecting CRE isolates is shown in Table 1. All the isolates were found to be resistant to meropenem, imipenem, ertapenem, ampicillin-sulbactam, piperacillin-tazobactam, cefazolin, cefuroxime, ceftriaxone, ceftazidime, cefepime, cefotetan, aztreonam, ciprofloxacin, levofloxacin, gentamicin, tobramycin and trimethoprim-sulfamethoxazole. All infecting CRE isolates except two *E. coli* were resistant to amikacin and tigecycline, while those infecting CRE isolates showed low resistance to colistin (5.6%).

**Table 1:** Antimicrobial resistance of subsequent infecting carbapenem-resistant Enterobacterale isolates

Antimicrobial agents	Number of isolates (%)	
	Resistance	Susceptibility
Meropenem	18 (100)	0 (0)
Imipenem	18 (100)	0 (0)
Ertapenem	18 (100)	0 (0)
Ampicillin-sulbactam	18 (100)	0 (0)
Piperacillin-tazobactam	18 (100)	0 (0)
Cefazolin	18 (100)	0 (0)
Cefuroxime	18 (100)	0 (0)
Ceftriaxone	18 (100)	0 (0)
Ceftazidime	18 (100)	0 (0)
Cefepime	18 (100)	0 (0)
Cefotetan	18 (100)	0 (0)
Aztreonam	18 (100)	0 (0)
Ciprofloxacin	18 (100)	0 (0)
Levofloxacin	18 (100)	0 (0)
Gentamicin	18 (100)	0 (0)
Tobramycin	18 (100)	0 (0)
Amikacin	16 (88.9)	2 (11.1)
Trimethoprim-sulfamethoxazole	18 (100)	0 (0)
Tigecyclin	16 (88.9)	2 (11.1)
Colistin	1 (5.6)	17 (94.4)

### Factors associated with subsequent CRE infection

Demographic and clinical characteristics of cases and controls are shown in Table 2. On univariable analysis, there was no significant difference in most comorbidities such as diabetes mellitus, solid tumor, pneumonia, liver disease, enteritis, gastritis, shock, or severe neutropenia between cases and controls. Patients who had admission to ICU, diarrhea, mechanical ventilation, or receiving PPIs were more likely to have subsequent CRE infection (*P* < 0.05) (Table 2). On logistic regression analysis, admission to ICU (OR, 15.087; 95%CI, 1.142–199.320; *P* = 0.039) and receiving PPIs (OR, 9.306; 95%CI, 1.015–85.341; *P* = 0.048) were associated factors for subsequent CRE clinical infection among HM patients with CRE rectal colonization (Table 3).

**Table 2:** Factors associated with subsequent CRE infection among hematological malignancies patients colonized with CRE

Variables	Case group (N = 18)	Control group (N = 36)	*P-*value
*Demographics*			
Age (Median, IQR)	38.5 (19–54.5)	40 (20–54.5)	
Sex, male	10 (55.6)	20 (55.6)	
*Hospitalization*			
Length of stay, (IQR,days)	52.5 (30.5–80.5)	49.0 (21.0–91.0)	0.783
Transferring from another hospital	3 (16.7)	8 (22.2)	0.905
Admission to ICU	6 (33.3)	2 (5.6)	0.021^*^
Prior hospitalization	13 (72.2)	23 (63.9)	0.540
*Comobidity conditions*			
Diabetes mellitus	2 (11.1)	3 (8.3)	1.000
Solid tumor	2 (11.1)	2 (5.6)	0.854
Pneumonia	14 (77.8)	18 (50.0)	0.050
Liver disease	9 (50.0)	10 (27.8)	0.107
Gastritis	4 (22.2)	2 (5.6)	0.168
Shock	8 (44.4)	9 (25.0)	0.147
Diarrhea	13 (72.2)	13 (36.1)	0.012^*^
	7 (38.9)	10 (27.8)	0.407
*Invasive procedures*			
Central venous catheter	14 (77.8)	30 (83.3)	0.901
Arterial catheter	4 (22.2)	2 (5.6)	0.168
Endotracheal intubation	2 (11.1)	3 (8.3)	1.000
Mechanical ventilation	10 (55.6)	8 (22.2)	0.014^*^
Urinary catheter	4 (22.2)	2 (5.6)	0.168
Nasogastric tube	4 (22.2)	1 (2.8)	0.068
HSCT	5 (27.8)	15 (41.7)	0.319
*Drug exposure*			
PPIs	15 (83.3)	18 (50.0)	0.018^*^
Glucocorticoid	13 (72.2)	19 (52.8)	0.170
Cephalosporins	6 (33.3)	4 (11.1)	0.107
Carbapenems	17 (94.4)	26 (72.2)	0.120
Fluoroquinolones	9 (50.0)	19 (52.8)	0.847
Glycopeptides	9 (50.0)	16 (44.4)	0.700
Tigecycline	8 (44.4)	11 (30.6)	0.314
Oxazolidones	6 (33.3)	10 (27.8)	0.673
Aminoglycosides	3 (16.7)	7 (19.4)	1.000
β-lactam/β-lactamase inhibitors	12 (66.7)	21 (58.3)	0.554
Macrolides	2 (11.1)	4 (11.1)	1.000
Antifungal agents	14 (77.8)	31 (86.1)	0.699
Antiviral agents	7 (38.9)	17 (47.2)	0.561
*CRE isolates*			
Klebsiella pneumoniae	10 (55.6)	17 (47.2)	0.564
Escherichia coli	6 (33.3)	9 (25.0)	0.519
Enterobacter cloacae	1 (5.6)	4 (11.1)	0.868
others	1 (5.6)	6(2.8)	0.64
*Types of hematological malignancy*			
AML	8 (44.4)	18 (50.0)	0.700
ALL	6 (33.3)	7 (19.4)	0.260
MM	0 (0.0)	2 (5.6)	0.313
Lymphoma	4 (22.2)	7 (19.4)	1.000
others	0 (0.0)	2 (5.6)	0.313

Values are presented as n(%), unless otherwise noted

OR, odds ration; CI, confidence interval; IQR, Interquartile range; ICU, intensive care unit; CRE, Carbapenem-resistant Enterobacterales; HSCT, hematopoietic stem cell transplantation; PPI, proton pump inhibitors; TMP/SMX, sulfamethoxazole and trimethoprim.; AML, Acute Myeloid Leukemia; ALL, Acute Lymphoblastic Leukemia; MM,Multiple Myeloma

^*^ Statistically significant differences between groups *(P* < 0.05)

**Table 3:** Logistic regression analysis of subsequent CRE infection among hematological malignancies patients colonized with CRE

Variables	OR (95%CI)	*P-*value
Admission to ICU	15.087 (1.142–199.320)	0.039^*^
Diarrhea	1.898 (0.460–7.829)	0.375
Mechanical ventilation	2.128 (0.511–8.865)	0.299
PPIs	9.306 (1.015–85.341)	0.048^*^

OR, odds ration; CI, confidence interval

^*^ Statistically significant differences between groups *(P* < 0.05)

### Mortality of subsequent CRE infection

**Fig. 2:**
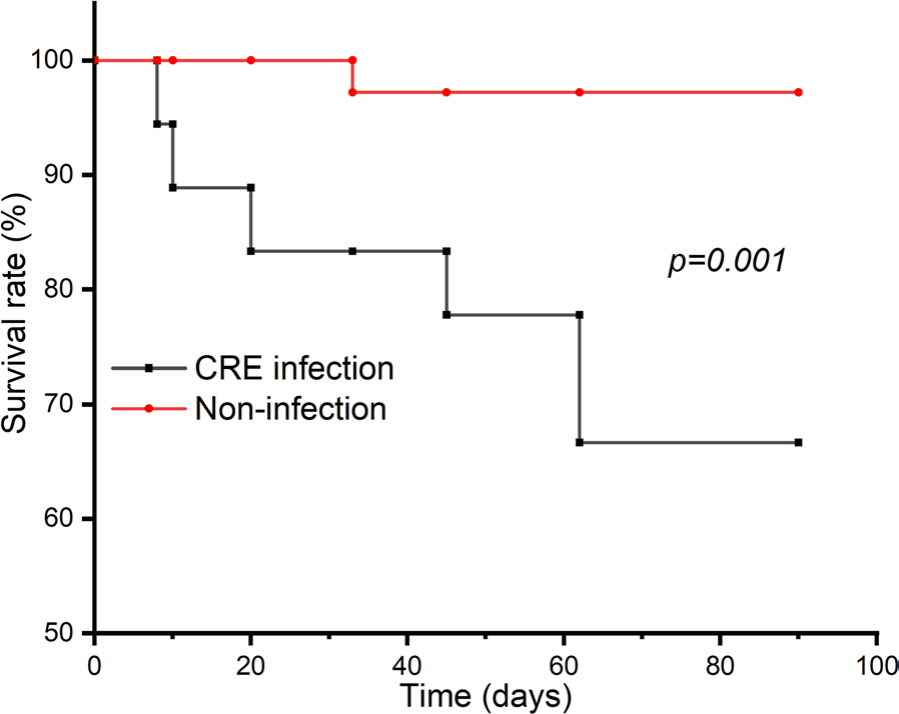
Kaplan–Meier survival estimates among hematological malignancies patients colonized with CRE

Seven (13.0%, 7/54) patients among the 54 colonized HM patients died during 90 days follow-up. Patients with subsequent CRE infection had higher mortality rate than that in controls (33.3%, 6/18 VS 2.78%, 1/36, *P* = 0.001) (Fig. 2). Of all the 18 subsequent CRE infection patients, 5 patients died of the bloodstream infection and 1 patient died of the pulmonary infection in 90 days follow-up were defined as the mortality group, other 12 patients alive in 90 days were classified as the survival group. The demographic and clinical characteristics of those two groups are shown in Table 4. On univariable analysis, shock showed significant difference between two groups (*P* < 0.05) (Table 4).

**Table 4:** Factors associated with the mortality of patients with subsequent CRE infection

Variables	Mortality group (N = 6)	Survival group (N = 12)	*P-*value
*Demographics*			
Age (X ± SD, years)	53.8 ± 24.2	31.4 ± 19.4	0.050
Sex, male	4 (66.7)	6 (50.0)	0.638
*Hospitalization*			
Length of stay, (IQR, days)	4 (66.7)	10 (83.3)	0.569
Transferring from another hospital	1 (16.7)	2 (16.7)	1.000
Admission to ICU	2 (33.3)	4 (33.3)	1.000
Prior hospitalization	5 (83.3)	8 (66.7)	0.615
*Comorbid conditions*			1.000
Diabetes mellitus	1 (16.7)	1 (8.3)	1.000
Solid tumor	1 (16.7)	1 (8.3)	1.000
Pneumonia	5 (83.3)	9 (75.0)	1.000
Liver disease	4 (66.7)	5 (41.7)	0.620
Gastritis	2 (33.3)	2 (16.7)	0.569
Shock	6 (100.0)	2 (16.7)	0.002^*^
Diarrhea	4 (66.7)	9 (75.0)	1.000
*Invasive procedures*			
Central venous catheter	4 (66.7)	10 (83.3)	0.569
Arterial catheter	2 (33.3)	2 (16.7)	0.569
Endotracheal intubation	2 (33.3)	0 (0.0)	0.098
Mechanical ventilation	4 (66.7)	6 (50.0)	0.638
Urinary catheter	2 (33.3)	2 (16.7)	0.569
Nasogastric tube	3 (50.0)	1 (8.3)	0.083
HSCT	0 (0.0)	5 (41.7)	0.114
*Drug exposure*			
PPIs	4 (66.7)	11 (91.7)	0.245
Glucocorticoid	5 (83.3)	8 (66.7)	0.615
Cephalosporins	1 (16.7)	5 (41.7)	0.600
Carbapenems	6 (100.0)	11 (91.7)	1.000
Fluoroquinolones	1 (16.7)	8 (66.7)	0.131
Glycopeptides	2 (33.3)	7 (58.3)	0.620
Tigecycline	2 (33.3)	6 (50.0)	0.638
Oxazolidones	1 (16.7)	5 (41.7)	0.600
Aminoglycosides	0 (0.0)	3 (25.0)	0.515
β-lactam/β-lactamase inhibitors	4 (66.7)	8 (66.7)	1.000
Macrolides	0 (0.0)	2 (16.7)	0.529
Antiviral agents	1 (16.7)	6 (50.0)	0.316
*CRE isolates*			
Klebsiella pneumoniae	5 (83.3)	5 (41.7)	0.240
Escherichia coli	1 (16.7)	5 (41.7)	0.596
Enterobacter cloacae	0 (0.0)	1 (8.3)	0.480
others	0 (0.0)	1 (8.3)	0.480
*Type of hematological malignancy*			
AML	1 (16.7)	7 (58.3)	0.240
ALL	2 (33.3)	4 (33.3)	1.000
Lymphoma	3 (50.0)	1 (8.3)	0.161
*Antimicrobial susceptibility profiles*			
Resistant to all the tested antimicrobial agents	0 (0.0)	1 (8.3)	1.000
Resistant to amikacin	6 (100.0)	10 (83.3)	0.529
Resistant to tigecyclin	6 (100.0)	10 (83.3)	1.000
Resistant to colistin	0 (0.0)	1 (8.3)	0.529
*Antimicrobial treatment*			
Carbapenems + amikacin	1 (16.7)	5 (41.7)	0.600
Carbapenems + tigecyclin	3 (50.0)	6 (50.0)	1.000
Carbapenems + colistin	1 (16.7)	2 (16.7)	1.000
Carbapenems + amikacin + tigecyclin	3 (50.0)	4 (33.3)	1.000
Carbapenems + tigecyclin + colistin	3 (50.0)	4 (33.3)	0.627
Carbapenems + amikacin + tigecyclin + colistin	1 (8.3)	0(0.0)	1.000

Values are presented as n(%), unless otherwise noted

OR, odds ration; CI, confidence interval; ICU, intensive care unit; CRE, Carbapenem-resistant Enterobacterales; HSCT, hematopoietic stem cell transplantation; PPI, proton pump inhibitors; TMP/SMX, sulfamethoxazole and trimethoprim; AML, Acute Myeloid Leukemia; ALL, Acute Lymphoblastic Leukemia

^*^ Statistically significant differences between groups *(P* < 0.05)

## Discussion

CRE has become an urgent public health issue worldwide for its high morbidity and mortality rate [28, 29]. Patients with hematological malignancies are at high risk of CRE infections for the immunocompromised state [3, 30]. The present study investigated the prevalence, factors and clinical outcomes associated with subsequent CRE infection among CRE colonized HM patients, and its impact on mortality. The results indicate that receiving PPIs and admission to ICU were important factors associated with subsequent CRE infection. In addition, shock was associated with high mortality among HM patients with subsequent CRE infection.

This study revealed 18.4% (18/98) HM patients colonized with CRE had subsequent CRE infection. The dominated subsequent infecting CRE species was *K. pneumoniae* due to it was also the mainly colonizing specie, which may lead to selection bias for CRE. Few studies are available regarding the prevalence of subsequent CRE infection following colonization focusing on this specific patient population. Regardless of patient population, Giannella et al. reported 7.8% carbapenem-resistant *Klebsiella pneumoniae* (CRKP) rectal carriers among all hospitalized patients developed a CRKP BSI after colonization, within a median of 19 days after the first positive rectal swab [31]. Amit et al. screened high risk patients from long-term care facilities, another acute care facility or abroad for rectal CRKP carriage, and found 19.7% patients developed BSI within 45 days of initial CRKP detection [32]. Our previous study showed that 37.1% patients developed subsequent infections among CRKP colonized patients, mainly in ICU patients [17]. The various infection rates observed among studies may due to different study population or types of infection.

This study showed receiving PPIs and admission to ICU were associated with subsequent CRE infection in HM patients with CRE rectal colonization (*P* < 0.05) though the CIs of OR showed a relatively wide range. These findings were different from two previous studies of colonized patient with hematological diseases in general. One study found gastrointestinal injury, tigecycline exposure and carbapenem resistance score were associated with subsequent CRE infection [15], while another study revealed that high-risk disease and mucositis were related to subsequent CRE infection [16]. The difference may be due to the different population. Those two studies did not focus on the patients with hematological malignancies.

Identifying receiving PPIs as an associated factor in this study is a novel and important finding. So far, there is no study available reported receiving PPIs associated with the risk of subsequent CRE infection among hematological patients with CRE colonization. A previous study confirmed that exposure to PPIs was significantly associated with infections derived from extended spectrum β-lactamase-producing enterobacteriaceae [33]. Studies have confirmed using PPIs can reduce gastric acidity and affect the gut microbiome more prominent than the effects of antibiotics [34]. PPI-induced changes of the microbiome may lead to clinical enteric or systemic infections [35, 36]. Previous studies showed that the risk of enteric infections in patients receiving PPIs were 2.5-fold greater than those without receiving PPIs [37]. PPIs are widely used in patients with malignancy. Patients with hematological malignancy who received PPIs may trigger bacterial translocation from the gut because of having immunosuppression and intestinal flora disturbance, a potential association between PPIs usage and development of gut-derived bacteraemia in hematological malignancy patients after chemotherapy [38]. This may explain why receiving PPIs is associated subsequent infection in HM patients with CRE colonization in this study.

Admission to ICU played an important role in subsequent infection among CRE colonized HM patients in this study. Similarly, Giannella et al. reported that admission to the Intensive Care Unit (ICU) was one of associated factors for CRKP BSI development among CRKP rectal carriers [31]. Chen et al. demonstrated patients had admission to ICU were more likely to develop CRE infection [17]. It's worth noting that admission to ICU may be a consequence of worsening clinical condition and weakened immunity which may increase the chances of infection. This finding suggests clinician should pay close attention to CRE colonized HM patients with admission to ICU to prevent subsequent infection.

In this study, the mortality of subsequent CRE infection was 33.3%, which is lower than that of HM patients with CRE bloodstream infection, ranging from 45.6%-100% [8–10]. This may be due to those patients were all with bloodstream infection while patients in this study included both bloodstream infection and pulmonary infection. Shock was an associated factor for mortality in this study. Similarly, shock has been reported associated with mortality of CRE infection in previous study [39].

This study has several limitations. First, it is a retrospective study, which has its inherent biases. Second, it is carried out in a single medical center and there may be some selection bias. Third, the molecular clonal relationship of the colonizing CRE strains and subsequent infecting strains was not confirmed although we only enrolled patients with subsequent CRE infection caused species as the colonizing CRE. Future molecular analysis should be performed to confirm this. Despite the limitations, this study is valuable for it detects the factors associated with subsequent infection in CRE rectal colonized patients with hematological malignancies for the first time.

## Conclusions

In conclusion, this study revealed *K. pneumoniae* was the dominate colonizing species and subsequent infecting species among HM patients with CRE colonization. Receiving proton pump inhibitors and admission to ICU increased the risk of subsequent CRE infection. For patients with subsequent infection, taking active action to control shock may improve clinical outcomes.

## Data Availability

The data used and/or analyzed in this study are available from the correspinding author on reasonable request.

## Abbreviations

CRE: Carbapenem-resistant Enterobacterales
HM: Hematological malignancies
ICU: Intensive care unit
PPIs: Proton pump inhibitors
MDR: Multidrug-resistant bacteria
GNB: Gram-negative bacteria
CRKP: Carbapenem-resistant *Klebsiella pneumoniae*
CRPA: Carbapenem-resistant *Pseudomonas aeruginosa*
